# The Hidden Burden of Trichinellosis in Vietnam: A Postoutbreak Epidemiological Study

**DOI:** 10.1155/2013/149890

**Published:** 2013-11-07

**Authors:** Nga Vu Thi, Dung Do Trung, Amber Litzroth, Nicolas Praet, Huong Nguyen Thu, Hien Nguyen Thu, Hung Nguyen Manh, Pierre Dorny

**Affiliations:** ^1^Parasitology Section, National Centre for Veterinary Diagnosis, 11/78 Giai Phong, Phuong Mai, Dong Da, Hanoi, Vietnam; ^2^Laboratory of Parasitology, Faculty of Veterinary Medicine, Ghent University, 133 Salisburylaan, 9820 Merelbeke, Belgium; ^3^Parasitology Department, National Institute of Malariology, Parasitology and Entomology, BC 10-200, Tuliem, Hanoi, Vietnam; ^4^Operational Directorate Public Health and Surveillance, Scientific Institute of Public Health, 14 J. Wytsmanstraat, 1050 Brussels, Belgium; ^5^Department of Biomedical Sciences, Institute of Tropical Medicine, 155 Nationalestraat, 2000 Antwerp, Belgium

## Abstract

A cross-sectional study was conducted in Muong Lat town (Thanh Hoa province, North Vietnam), following the confirmed diagnosis of trichinellosis in six patients from that town who had eaten hunted wild boar meat during the Vietnamese lunar year celebration. All inhabitants who declared to have eaten undercooked or raw wild boar meat at the celebration and showed at least one clinical symptom compatible with trichinellosis were included in the study and blood sampled. Anti-*Trichinella* IgG were determined by ELISA and Western Blot. Seropositive persons were given appropriate albendazole treatment and were followed up. A total of 100 inhabitants met the inclusion criteria. Among these, 30 (30%) had antibodies to *Trichinella*. Serologically confirmed cases had fever (90.0%), myalgia (86.7%), facial oedema (63.3%), diarrhoea (53.3%), and pain of the masseter muscles (43.3%). Eosinophilia was detected in 83.3% of these individuals. Clinical symptoms resolved in all patients during albendazole treatment. The results suggest that only a proportion of the trichinellosis cases had sought health care during the outbreak. There is a need to implement surveillance and better diagnosis for trichinellosis and to set up educational programs to prevent infection in North Vietnam.

## 1. Introduction

**Figure 1:**
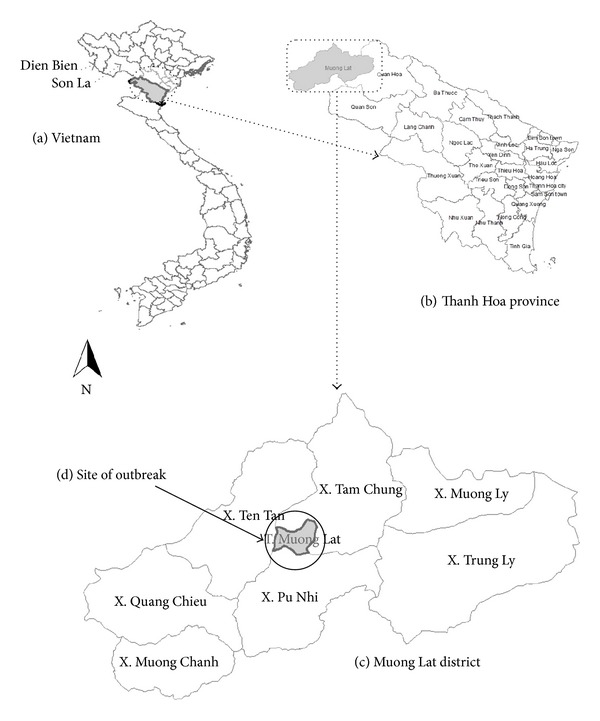
Map of Vietnam (a), Thanh Hoa province (b), Muong Lat district (c), with site of the outbreak (d).

Trichinellosis is a foodborne zoonotic disease [1, 2] that is commonly reported in Southeast Asia, especially in Thailand [3, 4] and Lao PDR [5–7]. During 1997–2012, five outbreaks of human trichinellosis were described in mountainous provinces of North Vietnam (i.e., Son La, Dien Bien and Thanh Hoa provinces; Figure 1) [8–10]. Diagnosis of the cases from these outbreaks was made in national hospitals many weeks after the onset because there is a lack of disease knowledge and facilities for diagnosis at provincial and district hospital levels [11].

On February 24, 2012, a cluster of 6 patients was hospitalized in Bach Mai hospital and the National Hospital for Tropical Diseases, Hanoi, with main clinical symptoms consisting of fever, muscular pain, difficult moving, facial oedema, and pain of the masseter muscles [10]. All patients tested positive in a *Trichinella* antibody ELISA and all cases showed positive muscle biopsy results for *Trichinella* larvae. The larvae were identified as *Trichinella spiralis *by molecular analysis. The patients' histories suggested that they all had consumed meat from a locally hunted wild boar during the Vietnamese lunar year celebration (Tet) in Muong Lat town (Muong Lat district, Thanh Hoa province; Figure 1), about one month before hospitalization in Hanoi. The objective of this study was to conduct a postoutbreak study in Muong Lat town shortly after diagnosis of trichinellosis in the 6 patients in order to determine if more inhabitants had nondiagnosed trichinellosis in this community. Ethical approval for the study was reviewed and approved by the Institutional Review Board of the Hanoi Medical University of the Vietnamese Ministry of Health (approval no. 100/HMU IRB).

## 2. Materials and Methods

### 2.1. Study Area and Sampling

The mountainous Muong Lat district (850 km^2^; 35959 inhabitants; 1 town and 7 communes) is located in Thanh Hoa province in the North Central Coast region of Vietnam, bordering Son La province, where an outbreak of human trichinellosis occurred in 2008, and Lao PDR (Figure 1). Muong Lat town, where the patients originated from, has a population of 3007 inhabitants, mainly of Kinh ethnicity, distributed over 543 households. All inhabitants of Muong Lat town were informed by the Muong Lat Medicine Centre about the study purpose before collecting samples. Inclusion criteria were (1) consumption of traditional dishes containing raw (*lap*) or undercooked (*nem chua*) meat prepared from a hunted wild boar during the previous Tet celebration and (2) presenting at least one of the symptoms that can be associated with trichinellosis (fever, facial oedema, myalgia, diarrhoea, etc.) as proposed by Dupouy-Camet et al. [12]. Individuals who met those criteria were blood sampled after giving informed consent. Structured questionnaires were used to collect information on the type of consumed meat, incubation period, and demographic information such as age, gender, and ethnicity. Giemsa-stained films prepared from human blood samples were preserved in boxes at room temperature until microscopic examination. Serum was collected from the blood samples by centrifugation and stored at −70°C until analysis. 

### 2.2. Laboratory Procedures and Data Analysis

Peripheral blood cell counts were performed by microscopy on Giemsa-stained films. A suspected case was defined as a patient with moderate (1000–3000 cells/*μ*L) to high (>3000 cells/*μ*L) eosinophilia [11]. Excretory/secretory (ES) antigen of *T. spiralis *first stage larvae, used in ELISA and Western Blot (WB), was produced using the protocol described by Gómez-Morales et al. [13]. Serum samples were first tested for the presence of anti-*Trichinella *IgG by ELISA using ES antigen [14, 15]. The cutoff of each plate was calculated based on the optical densities (OD) of 8 negative samples using a Student's *t*-test at a probability of *P* < 0.001. Positive controls were obtained from patients with confirmed trichinellosis, and negative controls consisted of serum samples from donors known to be *Trichinella *free. ELISA positive samples were tested by WB for confirmation [5, 13]. A pattern of three bands ranging in size between 48 and 72 kDa was considered to be positive [13]. Univariate and multivariate logistic regression analysis were used to investigate the relation between test seropositivity, age, and gender. Data analysis was performed using STATA/SE 11.0 (Stata Corp., College Station, TX, USA). The significance level was set at *P* < 0.05.

## 3. Results

A total of 100 individuals who consumed raw or undercooked hunted wild boar meat were identified as suspected cases of trichinellosis. Among these, the clinical symptoms observed were fever (60%), diarrhoea (41%), abdominal pain (40%), myalgia (37%), facial oedema (24%), and pain (20%). Gender distributions for the sampled individuals were women (*N* = 60, 60%)/male (*N* = 40, 40%); the median age was 31 years (range: 6–68 years); ethnicity was Kinh (*N* = 78, 78%) and minority groups (*N* = 22, 22%).

*Trichinella *antibodies were identified by ELISA in 30 samples of the 100 cases (30%). Of these 30 positive ELISA serum samples, all were also positive in WB. The most common clinical symptoms in these serologically positive patients were fever (*N* = 27, 90%), myalgia (*N* = 26, 87%), facial oedema (*N* = 19, 63%), diarrhoea (*N* = 16, 53%), pain of the masseter muscles (*N* = 13, 43%), and abdominal pain (*N* = 11, 37%). The median incubation period was 9 days (range: 4–17 days). A moderate to high eosinophilia was detected in 25/30 individuals (83.3%). The median age was 35 years (range: 6–60 years); ethnicity was Kinh (*N* = 20, 67%) and minority groups (*N* = 22, 33%). All 30 cases were orally treated with albendazole at a dosage of 15 mg/kg body weight per day for a period of two weeks during which they were clinically monitored by the Muong Lat Medicine Centre. Symptoms resolved in all patients during treatment. 

The multivariate analysis showed that the proportion of positive individuals was significantly higher in males (Odds Ratio (OR) = 3.18 (95% CI: 1.27–7.97); *P* < 0.05) and slightly increased with age (Odds Ratio (OR) = 1.04 (95% CI: 1.01–1.08); *P* < 0.05). The univariate analysis indicated the same significant associations.

## 4. Discussion

The results suggest that in Muong Lat town, where a trichinellosis outbreak occurred one month before this investigation, another 30 individuals who had eaten raw meat dishes prepared from the same wild boar had trichinellosis. Diagnosis was made based on clinical grounds, eosinophilia, and on ELISA followed by WB; no biopsies could be taken in this study for confirmation. Because anti-*Trichinella* IgG antibodies can persist for many years after infection/exposure to the parasite [16], it cannot be ruled out that the positive serological results were from older infections and not related to the current outbreak. However, the high proportion of seropositive results in the sampled patients strongly suggests an association with this outbreak. Several outbreaks of trichinellosis have been described recently in Northwest Vietnam [1, 9, 10]. Whether trichinellosis is an emerging infection in Vietnam or the identification of these outbreaks is rather the result of better diagnosis is not known. Trichinellosis is a disease primarily of adults, occurring about equally in both sexes; however, in some countries, among which Vietnam, infection in males occurs more frequently [1], which is consistent with our study.

Previous outbreak studies in Northwest Vietnam and in Lao PDR showed that pigs were the source of infection indicating the presence of a domestic life cycle [5, 17, 18]. The present study suggests that *Trichinella *spp. are also occurring in wildlife in Vietnam. Until now, the only species identified in the country is *T. spiralis*.

This study demonstrates the weakness of the diagnostic capacity and capability at provincial and district levels. It resulted in the underreporting of the number of patients infected during this outbreak, and it highlights the neglected characteristic of the infection, especially in remote rural areas where access to health care is often lacking. This study suggests that passive surveillance based on hospital records is likely to result in underestimating the real burden of trichinellosis due to the underreporting of the cases that did not reach the health care system. The study confirms that traditional dishes including raw *lap* and undercooked meat *nem chao *are to be considered as sources of infection. This outbreak emphasizes the need for education on the risks of acquiring this disease and the importance of thoroughly cooking meat. In addition, householders should be encouraged to adopt adequate livestock-breeding practices. Further study is recommended to investigate the presence of *Trichinella *in people and pigs in Thanh Hoa and neighbouring provinces.
